# Delivery and delay of guideline pharmacological, psychological, and social interventions for adults with complex psychosis in Dutch inpatient rehabilitation units: A retrospective study

**DOI:** 10.1177/00207640251358418

**Published:** 2025-09-03

**Authors:** Thijs J. Burger, Hans J. de Haas, Robin M. Van Eck, Martijn Kikkert, Frederike Schirmbeck, Astrid Vellinga, Lieuwe de Haan, Mariken B. de Koning

**Affiliations:** 1 Arkin, Institute for Mental Health, Amsterdam, The Netherlands; 2 Amsterdam University Medical Centre, Department of Psychiatry, Amsterdam, The Netherlands; 3 Central Institute of Mental Health, Department of Public Mental Health, Medical Faculty Mannheim, Heidelberg University, Mannheim, Germany

**Keywords:** complex psychosis, severe mental illness, inpatient mental health rehabilitation unit, instability of living environment, clozapine treatment, psychosocial interventions

## Abstract

**Background:**

People with complex psychosis admitted to inpatient mental health rehabilitation units (IMRU) constitute a low volume, high need, high cost group with a complicated recovery process.

**Aims:**

To generate hypotheses regarding successful delivery of guideline care to patients with complex psychosis in IMRUs, based on clinical profiles and (historical) patterns of care delivery.

**Method:**

A retrospective passive consent chart study of patients with complex psychosis in IMRUs in The Netherlands, mapping sociodemographic and clinical profiles, care trajectories and delivery of pharmacological (focusing on clozapine delay), psychological and social guideline interventions. We assessed relationships between non-delivery of psychological and social interventions, delay in clozapine treatment, and current symptom severity.

**Results:**

The 62 included patients had a mean illness duration of 21.6 years (*SD* 9.4); 89% were diagnosed with a schizophrenia spectrum disorder. They exhibited severe symptoms (GAF-s: mean 33, *SD* 12.2), substantial functional impairment (GAF-f: mean 32, *SD* 9.5), a history of physical violence (74%) and/or severe substance use problems (61%), and limited or absent insight (87%). Care trajectories showed long-term instability of living environment, and frequent current compulsory care (76%). Of 54 patients with a clozapine treatment indication, 65% currently used it, of whom 94% started in IMRU setting, and 43% had one or more earlier aborted trials. Support in developing day activities was accepted by 87% of total sample, including patients without a day activity goal on beforehand. 66% developed day activities. We found associations between symptom severity, clozapine delay, declining psychological treatment, and not having day activity goals.

**Conclusions:**

For some people with complex psychosis, clozapine treatment and psychosocial interventions may only materialize in specialized, long term, high structure settings offering continuity of care. Support in developing day activities may present a pathway to collaborative engagement. Early identification of those in need remains a challenge.

## Introduction

Deinstitutionalization, the replacement of long-stay psychiatric hospitals with smaller, less isolated community-based alternatives for the care of mentally ill people, has generally increased domestic and community living skills, freedom and quality of life for people with severe mental illness (Lamb & Bachrach, 2001; Leff & Trieman, 2000; Priebe et al., 2002). In The Netherlands, deinstitutionalization is relatively complete compared to other European countries by measure of key markers derived from care standards (Taylor Salisbury et al., 2016). Comprehensive services are in place for people with psychotic disorders, including assertive community based treatment and extensive supported housing programs. These housing solutions range from floating support services to residential care homes staffed 24 hr a day. Financial resources for mental health care have been clearly defined within the national budget, and the number of mental health professionals per 100,000 inhabitants is greater than 135.

Nevertheless, a small but high-cost subgroup of people with complex needs remains underserved in community mental health settings (Killaspy et al., 2016; Lamb, 1997). Often, they have experienced multiple unsuccessful attempts at supported or sheltered housing, which have led to harmful situations including severe social decline, violence, and contacts with the justice system (de Mooij et al., 2016). This may result in frequent and/or lengthy admissions. Although they present with a heterogeneous course and clinical picture, the general profile seen by clinicians is that of individuals with psychotic disorders that are hard to treat due to a lack of shared perspective on the problem and lack of response if treated, complicated by issues such as substance use, neurodevelopmental disorders, cognitive impairment, a low level of functioning, and importantly, an extensive/protracted history of serious harm to self and others associated with their psychiatric condition. A descriptive term used in the literature for this clinical picture is ‘complex psychosis’, introduced by Killaspy et al. (2016).

Although research focusing on this group of people is increasingly being conducted (and supports beneficial effect of long-term inpatient rehabilitation treatment), this group is still underrepresented in the scientific literature (Awara et al., 2023; Bunyan et al., 2016; van Kranenburg et al., 2019). Specifically, data on the nature (and possible deficiencies) of guideline-recommended treatment provided to them are scarce, and of special interest, given the complex, high need, high cost profile of this group, and the complicated recovery trajectories of the people within it (Burger et al., 2024; de Mooij et al., 2016; Whittle et al., 2024).

Of pharmacological interventions, the delivery (and delay) of clozapine treatment is particularly relevant in this context. Clozapine has unique efficacy in treatment-resistant schizophrenia (Kane et al., 1988; Tiihonen et al., 2017), and schizophrenia related substance use, suicidality, and aggression, which are often part of complex psychosis (Correll et al., 2022; Faden & Citrome, 2024; Rafizadeh et al., 2023). Also, clozapine prescription rates are known to vary and trend to underutilization and delay to initiation (Bachmann et al., 2017; Thien & O’Donoghue, 2019). Service user-centered guideline psychosocial interventions of special interest include vocational or day activity support and psychological treatment, which are well-regarded among service users (Dutch Psychiatric Association, 2012; National Institute for Health and Care Excellence, 2014, 2020; Wood et al., 2015).

The current study aimed to map the sociodemographic and clinical profile of people with complex psychosis treated in inpatient mental health rehabilitation units (IMRU) Amsterdam, The Netherlands and explore care trajectories and delivery of guideline interventions, to generate hypotheses regarding mechanisms behind non-delivery of guideline care to people with complex psychosis and ways to improve this matter. We registered the relative timing of key moments in medical treatment history, such as diagnosis, treatment resistance, long-term admission, and attempts at starting clozapine. We assessed the relationship between non-delivery of psychological and social interventions, (delay in) clozapine treatment, and current symptom severity. We hypothesized a positive relationship between these variables.

## Methods

### Study design and data collection

This is a retrospective chart study, with a passive consent procedure, within the context of inpatient mental health rehabilitation units (IMRU), at a large mental healthcare institution in Amsterdam, The Netherlands. IMRU are aimed at adults with complex psychosis, who after inpatient treatment in either acute clinics or high-security (including forensic) long-term inpatient services, which often has taken a year or more, are not yet considered ready to transition to community care.

IMRU deliver long-term treatment using recovery-oriented practice is delivered according to the Active Recovery Triad (ART) model, an established care model specific to the setting (Zomer et al., 2020, 2024). ART focuses on active (re)engagement of service users, their network, and professionals, to facilitate comprehensive recovery, ultimately enabling service users to reintegrate into the community. Operationalization of ART is supported by a model fidelity scale subdivided into domains such as team structure, organization of care, and architectural aspects of the facility, which are regularly audited. In IMRU, nursing care, social work, medical treatment, psychological treatment, individualized placement to day activities and/or supported jobs (‘day activity support’), and peer support by experts by experience are provided. Care intensity ranges from closed high-intensive care wards to apartments within or in proximity of the main facility. The maximum intended length of stay is 3 years, although this may be extended if after this period, community care arrangements are deemed still unfeasible.

The sample available to our exploratory study was small and our expectation based on clinical experience was it would be heterogeneous on multiple variables of interest. We aimed for a best as possible description of this group. Hence, we limited data acquisition by participants and time available only. Data collection ran from 1-5-2016 until 1-5-2019, at three distinct IMRU facilities in three different locations. All were part of Arkin mental health institution in Amsterdam, The Netherlands. During this period, MdK supervised data extraction by local clinical staff directly involved in treatment. At each IMRU, all inpatients ⩾18 years, who had been hospitalized ⩾1 year continuously in an inpatient setting per reference date (start of inclusion at the unit) were eligible for inclusion. For each participant, we manually extracted socio-demographic and clinical data based on extensive manualized chart review (including, but not limited to discharge letters and care plans), supplemented by treating clinician’s judgment. Data were pseudonymized and stored in a secure database.

#### Clinical outcomes

Symptom severity and functioning were rated by senior clinicians based on their familiarity with the case from standard clinical care, and entered into the database by junior clinical staff. We used the eight subscales of the Clinicians Rated Dimensions of Psychosis Symptom Severity (CRDPS, DSM-5). Each subscale is scored on a 0-4 scale, with 0 representing absent and 4 severe symptom severity (Ritsner et al., 2013). We added the Positive And Negative Symptom Scale (PANSS) hostility subscale, which is scored on a 1-7 scale, with 1 representing absent and 7 extreme severity of current psychopathology (Kay et al., 1987). We measured Global Assessment of Functioning (GAF) split for symptoms (GAF-s) and disabilities (GAF-f). Both are scored on a 1-100 scale, representing decreasing symptom severity and increasing levels of functioning, respectively. A GAF-s score of 1-10 is described as recurrent violence to others and/or self (including serious suicidal acts), a score of 90-100 as no symptoms. A GAF-f of 1-10 is described as persistent inability to maintain minimal personal hygiene, and 90-100 superior functioning in a wide range of activities (Pedersen & Karterud, 2012).

#### Guideline-recommended intervention characteristics

We documented the delivery of pharmacological (clozapine treatment), psychological (Cognitive Behavioral Therapy, CBT and/or Eye movement desentitization and reprocessing, EMDR), and day activity support interventions. We documented whether patients had an indication for clozapine treatment. Building on the Dutch guideline for clozapine use (2013), we defined clozapine indications as (i) treatment-resistant schizophrenia; (ii) treatment-resistant schizoaffective disorder, bipolar disorder, or depressive disorder with psychotic features; (iii) treatment-resistant substance abuse accompanying schizophrenia or schizoaffective disorder; (iv) treatment-resistant aggression accompanying schizophrenia or schizoaffective disorder; and (v) treatment-resistant aggression in autism or mental retardation with psychotic symptoms. We defined Psychosis-related treatment resistance as having had two other antipsychotics in adequate dosages for a minimum of 4 weeks and incomplete symptomatic recovery. Indications were scored according to abovementioned order; in cases where patients had multiple indications for clozapine, the one with the lowest number was registered. We defined delay to clozapine initiation as the time elapsed between the moment that two antipsychotics had been tried with insufficient effect on target symptoms and clozapine initiation. For current non-users, we determined delay of clozapine initiation to inclusion in the study, to investigate the duration of ongoing delay. We charted reasons for not using and/or discontinuing clozapine according to the clinician when a clozapine indication was present. To evaluate the efficacy of current clozapine treatment, we recorded whether patients had been transferred to a lower security level facility after start of clozapine and assessed clozapine resistance based on enduring target symptoms after clozapine initiation. For psychological interventions, we recorded lifetime psychological treatment and clinician-rated reasons for non-delivery. For day activity support we recorded having a day activity goal, acceptance of support, and actual development of day activities in the last 3 years.

### Statistical analysis

We presented *N* and % for categorical variables, mean and standard deviation (*SD*) for normally distributed continuous variables, and median and interquartile ranges (25%–75%) for non-normally distributed continuous variables. The following post-hoc analyses were undertaken. Using univariate regression and T-tests and η^2^ and Cohen’s d for effect size respectively, we explored the relationship between delay to clozapine initiation in current clozapine users with current total symptom severity (sum score of all CRDP subscales), having a day activity goal (yes/no), accepting support in developing day activities (yes/no) and not receiving psychological treatment due to decline of treatment offer or no subjective suffering (yes/no). To investigate the possible effect of inclusion bias, we ran sensitivity analyses excluding all patients that had been part of the informed consent pilot (see below). We used SPSS (version 28.0.1.1) for data management and statistics.

### Ethical considerations

The study protocol has been considered by the Institutional Review Board (IRB) and received an exemption from a full review, because patients in this study underwent no deviation from the established standard care, hence the project was not subject to the Dutch Medical Research Involving Human Subjects Act (Wet Medisch onderzoek, WMO). We hypothesized that an active informed consent approach would result in selection bias, so we tested this in a pilot study. Inpatients of a mental health rehabilitation unit were asked for consent to use their medical chart data for the study. Of fifteen patients, six declined to consent. Patients exhibiting severe paranoid symptoms were disproportionally represented among those who did not consent, indicating that an active informed consent procedure would introduce a considerable risk of selection bias. Consequently, a passive consent procedure was allowed by the IRB and implemented (Scholte et al., 2019). Patients and their legal guardians were informed about case file data use for research purposes and the possibility to opt-out, through regular newsletters distributed to patients of the IMRUs involved and their family/legal guardians.

## Results

### Sociodemographic and clinical characteristics

At the end of the inclusion period, 105 out of a total of 135 IMRU-admitted patients within the catchment area had been screened for eligibility. A total of 62 patients were enrolled in the study, including the nine patients who consented during the informed consent pilot-study. In all, 37 patients hospitalized <1 year and six patients who refused to participate during the informed consent pilot were excluded; no opt-outs occurred after the pilot phase.

Table 1 provides an overview of the sociodemographic and clinical characteristics of the sample. Patients were in their late 40s, predominantly male, and a sizeable minority was a first or second generation migrant. The majority had low education levels. Most patients had a primary diagnosis of schizophrenia spectrum disorder, while a smaller number had bipolar I disorder, or autism spectrum disorder with comorbid schizophrenia spectrum disorder, and comorbid substance use was frequent. GAF, CRDP, and PANSS subscale scores reflected severe symptoms and substantial functional impairment at inclusion. Most had a history of severe hostile behavior and of physical violence and/or a history of substance use. Lack of insight into the mental disorder was highly prevalent, both lifetime and in the last month.

**Table 1.** Sociodemographic and clinical characteristics of the total sample (*N* = 62).

Variable	*N*	%	*M*^a^/Median	*SD*^a^/25th–75th percentile
Male sex	35	56		
Age (*M* ± *SD*)			47.4	11.3
Level of education^b^				
Low	47	76		
Middle	8	13		
Higher	3	5		
Unknown	3	6		
1st or 2nd generation migrant	35	56		
Primary DSM IV/5 classification				
SSD	55	89		
Schizophrenia	37	60		
Schizoaffective disorder	14	23		
Other SSD	4	6		
Bipolar disorder	5	8		
ASD (comorbid SSD)	2	3		
Comorbid substance use disorder^c^	24	39		
GAF symptoms ( *M* ± *SD*)			33	12.2
GAF functioning (*M* ± *SD*)			32	9.5
Actual symptom severity (CRDP) (*M* ± *SD*)			13.6	5.2
Hallucinations (median, 25th–75th percentile)			2	1-4
Delusions (median, 25th–75th percentile)			3	1-4
Disorganization (median, 25th–75th percentile)			1	0-2
Abnormal psychomotor symptoms (median, 25th–75th percentile)			0	0-2
Negative symptoms (median, 25th–75th percentile)			2	2-3
Cognitive disabilities (median, 25th–75th percentile)			3	2-3
Depression (median, 25th–75th percentile)			1	0-2
Mania (median, 25th–75th percentile)			0	0-1
Actual hostility (PANSS; median, 25th–75th percentile)			3	1-3
History of hostility (PANSS; median, 25th–75th percentile)			7	6-7
History of violence to others				
None	4	7		
Verbal or directed at property	12	19		
Physical	46	74		
History of problems related to substance use^c^				
None	21	34		
Moderate	3	5		
Severe problems	38	61		
Insight in mental disorder during (most severe) episode				
Yes	1	2		
Limited	8	13		
Absent	53	85		
Insight in mental disorder past month				
Yes	8	13		
Limited	17	27		
Absent	37	60		

*Note*. ASD = Autism Spectrum Disorder; CDRP = Clinicians Rated Dimensions of Psychosis Symptom Severity; GAF = Global Assessment of Functioning; PANSS = Positive And Negative Syndrome Scale; SSD = Schizophrenia Spectrum Disorder.

a Mean or median reported: see after variable (between brackets).

b According to ISCED criteria.

c According to DSM-IV-TR criteria.

### Care trajectories, compulsory care, and competence

**Figure 1.**
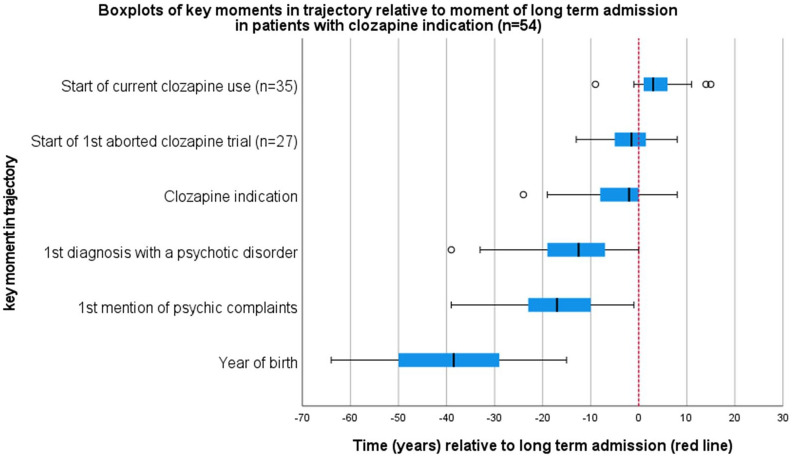
Boxplots of key moments in trajectory relative to moment of long-term admission. *Note*. Red line: moment of long-term admission.

Initial diagnosis with a psychotic disorder dated back over two decades on average. On average, time between diagnosis and establishment of treatment resistance was multiple years and this extended to over a decade. Treatment resistance preceded long-term admission by years on average, but was established after long-term admission in a quarter of cases. The long but heterogeneous periods between these transition points in treatment history indicate heterogeneous trajectories preceded IMRU admission (Figure 1, Table 2). Patients typically were in lengthy inpatient trajectories. They had made frequent transfers between different inpatient facilities and/or inpatient and outpatient setting in the last 10 years, indicating instability of living environment. A significant majority was involuntarily hospitalized and had a history of compulsory medication use, with some still under such treatment, indicating significant harm to self and/or others stemming from psychiatric disorder, and disagreement on the necessity of treatment. Most had a court appointed legal guardian or receiver, indicating high prevalence of incompetence to financial and/or other decisions, and opposition to accept support in these decisions. Legal guardians and receivers mostly had been appointed during the current admission. Table 2 provides an overview of care trajectories, compulsory care, and competence.

**Table 2.** Care trajectories, compulsory care, and competence.

Variable	*N*	%	*M*/Median^a^	*SD*/25th–75th percentile^a^
Duration^b^ since first documented mental health complaints or behavioral change (years, *M* ± *SD*)^c^	61		25.8	9.0
Duration^b^ since first diagnosis including psychotic symptoms (years, *M* ± *SD*)	62		21.6	9.4
Duration^b^ since therapy resistance established (years, median, 25th–75th percentile)^d^	54		11	7.75-17
Duration^b^ of uninterrupted inpatient care (years, median, 25th–75th percentile)	62		7	3-11
Time from first documented mental health complaints or behavioral change to first diagnosis including psychotic symptoms (median, 25th–75th percentile)^b^	61		2.0	0.0-8.0
Time from diagnosis including psychotic symptoms to treatment resistance established (years, median, 25th–75th percentile)^d^	54		8.5	4.75-13.0
Number of transfers last 10 years^e^ (years, median, 25th–75th percentile)	61		7	4-11
Current involuntary hospitalization	47	76%		
Current compulsory care for medication use	11	18%		
History of compulsory care for medication use	39	63%		
Support in financial management needed				
According to treatment team	58	94%		
According to patient	26	42%		
Has got voluntary budget management	11	18%		
Has got legal guardian or receiver	47	76%		
Legal guardian/receiver appointed during long-term admission	32	52%		

a Mean or median reported: see after variable (between brackets).

b Relative to inclusion date.

c 1 missing.

d Patients with therapy resistance established only (*n* = 54).

e Between inpatient and outpatient setting or between inpatient services.

### Delivery and delay of clozapine treatment

Table 3 provides detailed information on clozapine use within the patient sample. Figure 1 illustrates timing of clozapine trials relative to long-term admission. Most patients had an indication for clozapine use, and most were past or current users. We found a number of indicators of complicated clozapine commencement: Most patients currently treated with clozapine had started their current treatment only after long-term inpatient admission (Figure 1); 27 patients had one or more aborted clozapine trials: 16 one; 7 had two, 2 had three and 2 had four (also see Figure 1, 27/54: 15/35 currently on clozapine and 12/19 current non users). Most common reasons for ending these trials included non-compliance, patients withdrawing from treatment, and side effects. We observed that starting clozapine treatment was followed by transfers to lower security facilities and reductions in ward confinements for most (Table 3), indicating a clinically important reduction in harmful behavior. However, substantial symptom severity remained. In the patients currently not using clozapine, the primary barrier to starting or restarting clozapine was persistent patient refusal despite repeated counseling.

**Table 3.** Clozapine indication, prescription, and delay.

Variable	*N*	%	*M*/Median^a^	*SD*/25th–75th percentile^a^
Indication for clozapine (*n* = 54)				
Treatment resistant Schizophrenia	40	74%		
Treatment resistant schizo-affective disorder or bipolar disorder	11	20%		
Treatment resistant substance use disorder accompanying schizophrenia or schizo-affective disorder	2	4%		
Treatment resistant aggression in autism and mental retardation with psychotic symptoms	1	2%		
(History of) clozapine use in patients with an indication (*n* = 54)				
Indication + current use	35	65%		
Indication, history of use, no current use	12	19%		
Indication, no history of use	7	11%		
Delay to clozapine in ever users with an indication (*n* = 47; years)				
Time from diagnosis to first use (*n* = 45^b^; years, *M*, *SD*)			14.2	9.4
Time from therapy resistance to first use (*n* = 45^b^; years, median, 25th–75th percentile)			4	0.5-8.5
Time from therapy resistance to current use of clozapine in current users (*n* = 35; years, median, 25th–75th percentile)			6	1-10
Time from therapy resistance to inclusion date for never users (*n* = 7; years, median, 25th–75th percentile)			4	2-8
Treatment effect in current Clozapine users (*n* = 35)				
Reduction of restrictions in security level possible after start	30/35	86%		
Clozapine resistance	28/35	80%		
Indicators of complicated clozapine commencement in current users (*n* = 35)				
Number of antipsychotics used in history			3.8	1.7
History of one or more aborted trials with clozapine	15/35	43%		
Current clozapine use started during present admission	33/35	94%		
Time from present admission to start of current clozapine use			3	0-6
Current non-clozapine users with indication (*n* = 19)				
Patient’s refusal after repeated information	11/19	58%		
Serious side effects and/or non-response during previous attempt	5/19	26%		
Never considered by clinician	1/19	5%		
Unknown	2/19	11%		

a Mean or median reported: see after variable (between brackets).

b Excluding 2 patients for whom date of first attempt unknown.

### Delivery of psychosocial interventions

Table 4 shows delivery of psychological treatment and activity support. Large proportions of the sample did not have a psychological treatment goal or subjective suffering that would indicate psychological treatment, and/or did not have a day activity goal. Support in developing day activities was accepted by most patients. Notably, it was also acceptable to most without a goal (*n* = 21/29), half of whom indeed developed day activities (*n* = 12/21).

**Table 4.** Delivery of psychological and activity support guideline interventions.

Delivery of psychological treatment (lifetime, full sample (*n* = 62)		
Delivery of psychological treatment (CBT, EMDR)	21	39%
Reasons for non-delivery (more than one possible)		
No indication: no subjective suffering/treatment goal	32	48%
No indication: no coherent conversation possible	6	10%
Indication, but persistent decline of treatment offer	4	7%
Indication, but treatment not available	1	2%
Indication, started, premature termination by patient	3	5%
Indication, started, termination by professional due to no focus found	4	7%
Current or planned retrial of psychological treatment	7	12%
Delivery of day activity support in last 3 years (*n* = 62)		
Accepted support to develop day activities	54	87%
Has got a personal day activity goal	33	53%
Developed day activities		
Supported day activities	30	48%
Unsupported volunteer and paid work	11	18%

### Association between clozapine delay, non-delivery of psychosocial interventions, and symptom severity

In current clozapine users (*n* = 35), delay in starting current clozapine use significantly associated with current symptom severity with a large effect size (*F*(1, 33) = 8,810, *p* = .006, η^2^ = 0.211). In the total sample, 37 patients who had declined psychological treatment due to no treatment goal or subjective suffering (mean = 15.2 *SD* = 4.4) compared to the 25 other patients (mean = 11.1, *SD* = 5.3) had higher symptom severity (t(60) = 3.4, *p* = .001, d = 0.87), and 21 patients who did not develop day activities (mean = 15.4 *SD* = 5.9) compared to the 41 other patients (mean = 12.6, *SD* = 4.6) had higher symptom severity (t(60) = 2.1, *p* = .04. d = 0.56).

In current clozapine users (*n* = 35), 20 patients who had declined psychological treatment due to no treatment goal or subjective suffering (mean = 8.2, *SD* = 5.2) compared to the 15 other patients (mean = 4.4, *SD* = 5.6) trended to longer delays in starting current clozapine use (t(33) = 2.0, *p* = .05, d = 0.69). 16 patients without a day activity goal (mean = 8.8, *SD* = 5.9) compared to the other 19 patients (mean = 4.6, *SD* = 4.9) had longer delays in starting current clozapine use (t(33) = 2.3, *p* = .03, d = 0.78), whereas 23 patients who did develop day activities (mean = 7.9, *SD* = 4.4) compared to 12 patients who did not (mean = 5.8, *SD* 6.2) had no significantly longer delays (t(33) = 1.0, *p* = 0.31).

## Discussion

The current study intended to gain insight in the clinical profile, care trajectories, and delivery of guideline-based interventions of people with complex psychosis requiring IMRU care in The Netherlands. Specifically, we explored delivery of clozapine treatment (and its delay), psychological treatment and day activity support, their interrelation, and their relationship with present symptom severity. Our findings provide a basis to hypothesize on mechanisms behind (non) delivery of guideline interventions in this group and on ways to improve on this matter.

### A paradox of high needs and non-delivery of interventions

The clinical profile of IMRU patients indicated complex disorder and needs, with long-term difficulties in collaboration and recovery. The vast majority had a long history of psychotic disorder with severe current symptomatic burden and functional impairment, compounded by comorbid substance abuse, hostile behavior, history of physical violence, and/or lack of insight. The level of symptomatic and functional impairments exceeded the severity of disorder reported in a recent Nation-wide English inpatient rehabilitation service sample, especially concerning level of functioning and challenging/hostile/violent behaviors (Killaspy et al., 2016). Care trajectories in our sample showed long-term instability of living environment, opposition to treatment, and incompetence combined with opposition to support in decision making.

Indications for clozapine use were much more prevalent in our sample admitted to IMRU compared to a Dutch community care sample (87% vs. 33.4%), while clozapine use among those with an indication was similar to the current sample (65% vs. 68.8%) (van der Zalm et al., 2018). Although symptomatic remission was uncommon – an outcome consistent with previous findings in people with chronic psychotic illness (Kane et al., 2001) – the treatment led to a reduced need for security measures. This represents a highly clinically relevant improvement and may be taken as a marker of improved collaboration in multiple life areas, such as accepting support in daily life tasks or maintaining relationships with family and professionals.

We observed a paradox in patients admitted to IMRU: high severity of disorder and harm to self/others that *in theory* warrants combined use of guideline interventions; and *in practice*, non-delivery of those interventions. Current clozapine treatment typically only started after long-term admission, and past aborted trials were common, much more than in a Dutch community-based sample (van der Zalm et al., 2018). The resulting delays to current clozapine treatment in IMRU patients are at the longer end of delays known from literature (Thien & O’Donoghue, 2019). Psychological treatment (CBT, EMDR) and development of day activities often failed to materialize.

The paradox of non-delivery of interventions in those that need them most also holds *within* our sample. Higher present symptom severity associated with clozapine delay, non-delivery of psychological treatment, and not developing day activities. Non-delivery of the three interventions associated with each other. All in all, our findings parallel the pattern found outside long term inpatient settings (Griffiths et al., 2021; Shah et al., 2018).

### Ways to intervention delivery in complex psychosis

The following profile factors of IMRU patients may contribute to non-delivery of guideline interventions in this group: Low educational attainment, high substance abuse, and high symptom severity with low insight. These have been known to limit the effectiveness of pharmacotherapy, psychological treatment, support in developing day activities, and outcome, and are to be taken into account when designing interventions for this group (Carbon & Correll, 2014; de Winter et al., 2022; O’Keeffe et al., 2017). Based on our findings, we suggest a focus on developing day activities as an entry to collaboration, as even in patients without a goal, support was accepted and could lead to development of day activities.

Furthermore, the successful initiation of clozapine treatment only after long-term admission and often after earlier failed attempts, suggests that the IMRU environment is needed for delivery of treatment to some, as has been stressed in long existing discourse (Killaspy & Dalton-Locke, 2023; Lamb, 1997).

We hypothesize that the following characteristics of IMRU may be essential: (a) IMRU provides a highly structured environment that includes the possibility to use assertive and compulsory care (including locked doors and intensive supervision of adherence to pharmacotherapy), and access to professionals with special expertise in treating complex psychoses (Bogers et al., 2016). This may provide the conditions needed to limit service disengagement and care discontinuity due to harmful incidents; (b) IMRU provides a long-term stay, breaking the pattern of fragmentation found in our study. It provides stability of living situation and long-term care contact, which may facilitate development of a working relationship with professionals (where the baseline may be a hostile stance) and re-engagement of the network. We primarily point to IMRU as a way of delivering a long-term structured environment. This may be especially relevant in the contemporary context of outreaching outpatient services and sheltered housing facilities often lacking the resources needed to match the care needs of the people who encounter the most complex problems. A growing body of evidence shows that in moments (or long term situations) of vulnerability, people need long-term relationships and relational care to build on. Professionals are motivated to provide such care, but are hindered by health services/systems that prioritize short-term care trajectories and problem- and/or individual-based, rather than strength and/or network-based approaches (Burger et al., 2024; Muusse et al., 2020, 2021; Zomer et al., 2020). Bringing together the multiple resources that are needed within one service, on one location, may limit logistic problems, amplify their compound effect, safeguard their availability, and foster growth.

Incorporating highly structured treatment such as IMRU in early intervention services may prevent some very cumbersome trajectories. We suggest that the clinical profile of current research, a *late-stage* profile, may inform timely case selection: it mimics the *baseline* profile found to predict unfavorable outcome (i.e., non-remission of symptoms, substance abuse, low insight/treatment engagement, low functioning level, and recurrent admissions) in prospective studies, including individualized prediction modeling (de Nijs et al., 2021; Lambert et al., 2010; van Dee et al., 2023). However, the challenge to identify those in need at an early stage still remains: some with an unfavorable outlook may recover without intensive treatment, some may develop treatment resistance at a later stage only (Lally et al., 2016). This may be reflected by the heterogeneous trajectories that preceded IMRU admission in our sample. It remains crucial to balance possible benefits of IMRU treatment against the risk of overprotection that effectively limits recovery.

### Strengths and limitations

An important strength of this study is that we mapped a low volume, high need, high cost group with a complex clinical picture, not the strictly defined clinical syndrome usually prioritized in research (Taipale et al., 2022). We followed a passive informed consent procedure, thereby minimizing the risk of inclusion bias in this challenging population (de Koning et al., 2024) and we provided a high retrospective resolution on evidence based treatment delivery. Study limitations are its retrospective design, the fact that the data quality depended on clarity and comprehensiveness of historical clinical notes made by multiple clinicians, and manual data extraction by multiple raters between facilities (we did not assess inter-rater reliability). Moreover, the study had a pragmatic design and comparative analyses have been added post-hoc, precluding sample size calculations. Lastly, the guideline interventions investigated here were limited to clozapine and psychological treatment, and day activity support. Delivery of family interventions was not included in our dataset, while its general effectiveness has been established (Camacho-Gomez & Castellvi, 2020), and we did not investigate other evidence-based medical interventions (e.g., mood stabilizers, electroconvulsive therapy). Lastly, the transportability of our findings to other healthcare contexts hinges on service availability and accessibility: the population of interest might also reside on streets and/or in the justice system (Chow & Priebe, 2016).

## Conclusion

The current study shows the challenges of delivering guideline pharmacological (clozapine), psychological, and social interventions in adults with complex psychosis admitted to inpatient mental health rehabilitation units. We hypothesize that in this group with a high vulnerability profile and complex needs, offering too little structure and continuity can lead to a protracted lack of shared recovery objectives, fragmented care patterns, and non-delivery of evidence-based interventions. For some people with complex psychosis, clozapine treatment may only materialize in specialized, long term, high structure settings. Support in developing day activities may present a pathway to collaborative engagement. The challenge of timely identification of those in need remains.

## Supplemental Material

sj-docx-1-isp-10.1177_00207640251358418 – Supplemental material for Delivery and delay of guideline pharmacological, psychological, and social interventions for adults with complex psychosis in Dutch inpatient rehabilitation units: A retrospective study Supplemental material, sj-docx-1-isp-10.1177_00207640251358418 for Delivery and delay of guideline pharmacological, psychological, and social interventions for adults with complex psychosis in Dutch inpatient rehabilitation units: A retrospective study by Thijs J. Burger, Hans J. de Haas, Robin M. Van Eck, Martijn Kikkert, Frederike Schirmbeck, Astrid Vellinga, Lieuwe de Haan and Mariken B. de Koning in International Journal of Social Psychiatry
