# Supplementary material for: Delivery and delay of guideline pharmacological, psychological, and social interventions for adults with complex psychosis in Dutch inpatient rehabilitation units: A retrospective study
Source: Int J Soc Psychiatry. 2025 Sep 3;72(2):269–80. doi: 10.1177/00207640251358418 (PMC12946213; doi:10.1177/00207640251358418)
Supplement: sj-docx-1-isp-10.1177_00207640251358418 – Supplemental material for Delivery and delay of guideline pharmacological, psychological, and social interventions for adults with complex psychosis in Dutch inpatient rehabilitation units: A retrospective study [file sj-docx-1-isp-10.1177_00207640251358418.docx]

Research Protocol

## Contact details

| Title | Therapy-resistance and Recovery in Amsterdam Inpatients in  Long-term psychiatric hospitals (TRAIL) |
| --- | --- |
| Date | 17-12-2015 |
| Version | 4 |
| Contact | Dr. Burger, T.J. |
| Primary investigator | Dr. M.B. De Koning |
| Principal investigator | Dr. M.B. de Koning (Arkin, research department)  Prof. Dr. J. Dekker (Arkin, research department)  Prof Dr. L. de Haan (Academic Medical Center, Arkin, Academic Working Group SPD) |
| Sponsor | Arkin mental health institute |

## Study details

| Rationale | Clinical practice in social psychiatry shows that there is a small group of patients with a severe mental illness (SMI) who are very difficult to treat in outpatient settings. These patients reside in long-term clinical settings, often after a lengthy clinical and/or outpatient history, and often after multiple unsuccessful attempts at independent or supported living. Although these patients often function poorly and healthcare costs for this group are high, little research has been conducted on them. These patients often (but not always) have a psychotic disorder as the primary diagnosis and have frequently posed a danger to themselves or others in outpatient settings (including supported living) or previous clinical settings. So far, treatment has not been sufficiently effective to allow discharge from the clinic. Three themes are important when studying this patient group: ‘treatment resistance,’ ‘quality of life,’ and ‘recovery.’ In the TRAIL study, the profile of the group described above will be determined, and the types of treatments used biologically, psychologically, and socially will be investigated. \| |
| --- | --- |
| Objective | The primary objective of this study is to determine the profile of a group of long-term admitted patients in terms of history, (comorbid) diagnosis, applied and current treatment offered biologically, psychologically, socially, and legally (required amount of coercion/compulsion). |
| Study design | This is a retrospective observational study based on patient records.  There is no patient contact involved. |
| Study population | The target group consists of patients with an uninterrupted clinical admission longer than 1 year who meet the definition of Severe Psychiatric Disorder (SPD) patient.  Patients will be included from the following facilities at Mentrum, inpatient mental health rehabilitation facilities, part of Arkin: “Sporenburg”; “Reigersbos”; “Jan Thomeepad”; “Jean Desmetstraat” and Inforsa, part of Arkin: “Langdurige intensieve zorg”. |
| Inclusion criteria | 1. Admitted to a psychiatric clinic for more than 1 year; 2. Meets the definition of severe psychiatric disorder (SPD) by Delespaul (2013, Journal of Psychiatry): – presence of a psychiatric disorder requiring care/treatment (not in symptomatic remission); – accompanied by severe limitations in social and/or societal functioning (not in functional remission); – the limitation is both cause and effect of the psychiatric disorder; – not temporary (structural or long-lasting, at least several years); – coordinated care by professional caregivers within care networks is indicated to realize the treatment plan. \| |
| Exclusion criteria | none |
| Sample size | This is observational research, primarily descriptive statistics will be used. Calculating a required sample size for this type of question with an ordinal categorical outcome variable is complex and requires assumptions that are partly arbitrary. It is estimated that a group of 150 patients is large enough to demonstrate significant and clinically relevant correlations. |
| Recruitment | his concerns dossier research in a patient population in which it has been found that an informed consent procedure leads to significant selection bias due to refusal of participation by some patients because of paranoia. No informed consent will be requested; in principle, all patients admitted to the participating clinics during the study period will be included. Patients and their legal representatives will be informed via a newsletter about the coded use of their data for research purposes and the possibility to opt out without giving reasons. Patients and their legal representatives who refused participation after informed consent was asked in pilot studies will not be included.  Patiënten en hun wettelijk vertegenwoordigers waaraan in pilot-onderzoek informed consent is gevraagd en die deelname weigerden, zullen *niet* worden geïncludeerd in het onderzoek. |
| Intervention | None, only case file research |
| Study endpoints | Profile characteristics of patients who are long-term admitted in a clinical setting, including current and historical treatment methods used. |
| Study parameters | 1. Clinician-rated dimensions of psychosis symptom severity according to DSM-5 2. Stage according to DSM-5 3. Profile characteristics, according to the Academic Workgroup SPD Arkin supplemented with specific scoring instructions for the studied patient group 4. Additional dossier parameters concerning current functioning; current and used treatment (biological, psychological, social, legal), contributing factors to recovery. |
| Statistical analysis | Descriptive statistics |
| Subject burden | Case file research, no informed consent procedure, no burden for subjects. |
| Subject risks | none |
| Participation benefits | none |
| Disadvantages of participation | none |
| Participant compensation | n/a |
| Administrative aspects | Information is extracted and coded from patient records by members of the treatment team (psychiatrist, physician assistant, psychologist, psychologist in training). The information is then processed into a digital database. Coded dossier information is securely stored digitally within the institution for 15 years. Research data can only be traced back to individuals through the research number. The key is managed by a research staff member within institution not involved in study |
| Publication policy and amendments | The aim is to publish results of this study in at least two international peer-reviewed journals. Research results in these publications will not be traceable to individuals but will contain only group averages |
| Other | The study described above will be followed as planned by a study on experienced quality of life and recovery as reported by the patient, relatives, and caregivers. A separate non-WMO application will be submitted for this. |
